# Effect of Temperature and Water Content on the Physicochemical Properties of Crude Lecithin and Soybean Oil: A Response Surface Methodology Approach

**DOI:** 10.3390/molecules31101750

**Published:** 2026-05-20

**Authors:** Toktam Mohammadi-Moghaddam, Hamid Bakhshabadi, Afsaneh Morshedi, Seyed Mehdi Hosseini, Marcos E. Valdes

**Affiliations:** 1 Workplace Health Research Center, Neyshabur University of Medical Sciences, Neyshabur 170518, Iran; mohammadit1@nums.ac.ir; 2 Healthy Ageing Research Centre, Neyshabur University of Medical Sciences, Neyshabur 170518, Iran; 3 Department of Agriculture, Minab Higher Education Center, University of Hormozgan, Bandar Abbas 7916193145, Iran; h.bakhshabadi@hormozgan.ac.ir; 4 Noncommunicable Diseases Research Center, Neyshabur University of Medical Sciences, Neyshabur 170518, Iran; 5 Khorasan Cotton and Oilseeds Company, Neyshabur 9331143317, Iran; 6 Faculty of Gastronomic Sciences and Tourism, Universidad UTE, Quito 170508, Ecuador

**Keywords:** soybean oil, lecithin, oxidative parameters, industrial scale, response surface methodology, process optimization

## Abstract

In industrial soybean lecithin production, process variables such as temperature, water content, agitation, and processing time play a critical role in determining the final product quality. Despite their importance, the combined effects of these parameters under industrial-scale conditions have not been sufficiently quantified. Therefore, this study systematically evaluated the influence of processing temperature (70–80 °C) and water content (1.5–3% *w*/*w* of oil) on key quality attributes, including lecithin moisture content, purity, acidy number, and peroxide value, as well as soybean oil acidity and insoluble fine substances. A response surface methodology (RSM) approach was applied to model the relationships between variables and to identify optimal processing conditions. The results indicate that an increasing temperature reduces lecithin moisture content, likely due to enhanced water evaporation and improved phase separation, while simultaneously increasing peroxide value and oil acidity (*p* < 0.05), possibly because of accelerated lipid oxidation and hydrolysis. Lecithin purity improved up to 75 °C, indicating more efficient separation, but decreased at higher temperatures, suggesting thermal degradation or emulsification effects. Similarly, increasing water content led to higher moisture, peroxide value, and acidy numberin lecithin, as well as increased acidity and insoluble fine substances in soybean oil (*p* < 0.05), which can be attributed to excessive hydration promoting emulsion stability and entrainment of oil-phase impurities into the lecithin fraction. Process optimization indicated that a water content of 1.5% and a temperature of 73.93 °C yielded the highest overall product quality, with a desirability value of 0.874.

## 1. Introduction

Lecithin is one of the major by-products of the vegetable oil industry, particularly soybean oil processing, and is widely used in both food and non-food applications [1,2]. Chemically, lecithin consists of a complex mixture of glycolipids, triglycerides, and phospholipids, being mainly composed of phospholipids (phosphatidylinositol, phosphatidylcholine, and phosphatidylethanolamine) [2,3]. It is a viscus and sticky substance that ranges in color from yellow to brown, with a liquid or plastic-like consistency depending on the source of raw material, processing conditions, and subsequent treatments such as filtration or bleaching [4,5].

Soybeans (*Glycine max*) are one of the most important oilseed crops worldwide, containing approximately 40% protein, 23% carbohydrates, 20% oil, 5% minerals, 4% fiber, and 8% moisture [6]. Lecithin is naturally present in several food sources, including soybeans, eggs, milk, sunflower, rice, rapeseed, and canola. Among these sources, soybean lecithin is the predominant commercial form, representing approximately 0.3–0.6% of total oilseed weight. This highlights the importance of soybean processing conditions in maximizing lecithin yield and ensuring product quality [7].

Due to its multifunctional properties, lecithin plays a crucial role in the food industry. It is widely used as an emulsifier, wetting agent, crystallization inhibitor, viscosity reducer, and release agent [8]. Consequently, lecithin is incorporated into a wide range of food products including margarine, bakery items, confectionary, chocolate, noodles, shortenings, powdered milk, ice cream, instant coffee, beverages, desserts, sauces, and soups [9]. In addition to its technological functions, lecithin also exhibits antioxidant properties and is a crystallization inhibitor in fats and oils [3,7,8,9,10,11,12]. These versatile applications have contributed to the rapid expansion of the global lecithin market, which reached an estimated value of USD 1.59 billion by the end of 2023. Between 2018 and early 2023, global demand increased at an average annual growth rate of approximately 7% [10].

From a nutritional and formulation perspective, modern food product development increasingly focuses on reducing oil content to address public health concerns, such as obesity and cardiovascular disease. One effective strategy involves incorporating water into oil-based systems in the form of water-in-oil (W/O) emulsions, which enables fat reduction while maintaining desirable textural and sensory properties [2]. In such systems, lecithin plays a critical role as a natural emulsifier due to its amphiphilic structure, which facilitates the stabilization of dispersed water droplets within the oil phase.

Lecithin can be extracted using organic solvents, yielding a product suitable for human consumption, and is commercially available in various physical forms, including liquids, plastic-like masses, and free-flowing powders [3,7,8,9,11].

Importantly, the functional properties of lecithin—such as its emulsifying capacity, viscosity, and stability—are strongly influenced by its composition and processing history.

In industrial practice, lecithin recovery during vegetable oil refining involves the hydration of phosphatides using controlled amounts of water or steam, followed by centrifugation to separate the hydrated phospholipids from the oil phase, and subsequent drying of the lecithin precipitate. The efficiency of this process, as well as the quality of the final lecithin, is highly dependent on processing parameters such as temperature and water addition, which govern the physicochemical interactions between oil, water, and phospholipids [6,12,13].

From a mechanistic perspective, lecithin recovery during oil hydration is governed by complex process–structure–property relationships. The addition of water induces the hydration of phospholipids, leading to swelling, aggregation, and eventual phase separation due to their amphiphilic structure. Processing parameters such as temperature and water content directly influence intermolecular interactions, including hydrogen bonding, polarity changes, and micelle or lamellar structure formation. These structural transformations significantly affect the efficiency of phase separation, as well as the physicochemical properties of both lecithin (e.g., moisture, purity, oxidation stability) and the residual oil phase. Similar process–structure–property frameworks have been widely emphasized in recent food processing studies, where pretreatment and extraction conditions are shown to control microstructure evolution and final product functionality [8,12]. Therefore, a systematic and statistically driven approach is essential to understand and optimize these interdependent effects [14,15].

Although numerous studies have investigated lecithin production and extraction processes, recent advances in food processing research emphasize the importance of integrating mechanistic understanding with process optimization to control structure and functionality.

Key processing parameters such as temperature, water content, agitation intensity, and residence time play critical roles in determining the physicochemical properties of the recovered lecithin. Variations in these parameters can markedly affect product characteristics. For example, higher water levels and longer processing times may lead to darker lecithin, whereas insufficient processing or lower temperatures may result in incomplete phosphatide hydration and residual oil in the final product [13,14]. In industrial practice, typical conditions involve the addition of approximately 2% water (based on oil volume), processing times of 0.5–1 h, and temperatures ranging between 70 and 90 °C [13].

Despite extensive research on lecithin production and extraction processes, there remains a lack of studies that integrate industrial-scale conditions, mechanistic insights, and statistical optimization tools such as response surface methodology (RSM) to systematically evaluate the combined effects of processing variables [13,15,16,17,18,19,20]. Addressing this gap is essential for improving process efficiency, product consistency, and industrial scalability.

Therefore, the objective of the present study was to systematically investigate the effects of key hydration parameters—specifically, inlet soybean oil temperature (70, 75, and 80 °C) and water content (1.5%, 2.25%, and 3% *w*/*w*)—on the physicochemical properties of lecithin (moisture content, purity, peroxide value, and acidy number) and soybean oil (acidity and oil-insoluble fine substances). The selected parameter ranges were defined based on extensive industrial experience in oil extraction and lecithin production from various oilseeds, ensuring their practical relevance to commercial operations.

To quantitatively evaluate and optimize these processing conditions, response surface methodology (RSM) coupled with a central composite rotatable design (CCRD) was employed. This approach enabled the development of predictive models and the identification of optimal operational conditions for maximizing lecithin and oil quality under industrial processing constraints.

## 2. Results and Discussion

Table 1, Table 2 and Table 3 present the model selection for the dependent variables, the analysis of variance (ANOVA) used to determine the parameters, and the regression equations developed for the dependent variables, respectively.

### 2.1. Physicochemical Properties of Lecithin

#### 2.1.1. Moisture Content

**Figure 1:**
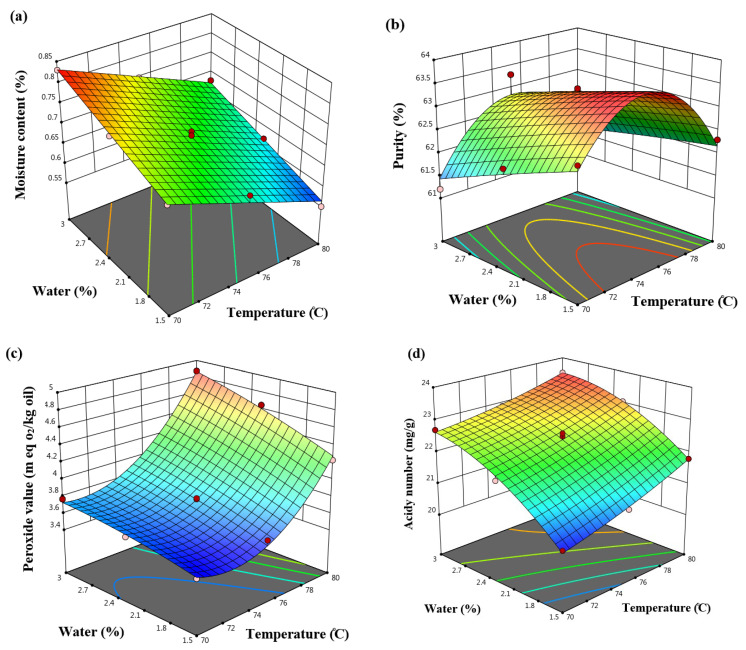
The effect of water and temperature on the (**a**) moisture content, (**b**) purity, (**c**) peroxide value, and (**d**) acidy number of lecithin.

The linear model was identified as the best fit for the moisture content data (*p* < 0.0001) (Table 1). Analysis of variance further indicated that the linear effects of water content and temperature significantly influenced the moisture content of the oils (*p* < 0.0001) (Table 2). Results showed that increasing the temperature decreased the moisture content, whereas higher water addition increased it (Table 3; Figure 1a). The moisture content of lecithin in this study ranged from 0.55% to 0.83%.

Srinuan et al. (2020) [21] investigated lecithin extraction from soybean using various solvents and a water-degumming process. They reported ethanol as the most effective solvent and found that the phosphatidylcholine content in commercial soybean lecithin was lower than in the extracted lecithin. The moisture content of lecithin in their study (5.4–6.8%) was notably higher than that observed in the present work, likely due to differences in soybean variety and solvent type used for oil extraction.

Previous studies have shown that higher water content during gum separation complicates lecithin recovery, leaving more residual water in the lecithin [22]. Moreover, excessive water addition decreases final oil yield, as more oil is transferred into the lecithin phase [23,24]. The reduction in moisture content at higher temperatures can be attributed to increased evaporation of water and reduced stability of the water–oil emulsion, which facilitates water separation during processing [25].

#### 2.1.2. Purity

Table 1 indicates that the quadratic model provided the best fit for describing the effects of process variables on lecithin purity. Analysis of variance (Table 2) showed that the linear effect of water addition and the quadratic effect of temperature significantly influenced purity (*p* < 0.005), confirming the presence of nonlinear interactions between processing parameters. Furthermore, the model coefficients (Table 3) revealed that temperature had a more pronounced effect on purity compared to the other variables.

The observed decrease in lecithin purity with increasing water content can be attributed to the enhanced hydration of phospholipids, which promotes the co-extraction of non-phospholipid components, such as insoluble fine particles and other minor constituents, into the lecithin phase [13]. This result highlights the sensitivity of phase selectivity to water dosage during the hydration process.

Temperature exhibited a nonlinear effect on lecithin purity. Increasing the temperature up to 75 °C improved purity, likely due to reduced oil viscosity and enhanced mass transfer, which facilitate more efficient aggregation and separation of hydrated phospholipids. However, further temperature increase (75–80 °C) resulted in a decline in purity. This behavior may be associated with structural alterations of phospholipids, such as partial denaturation or disruption of organized assemblies, which can hinder effective phase separation. In addition, higher temperatures may promote the formation of free fatty acids and other degradation products, negatively affecting lecithin quality [13]. These findings are consistent with process–structure–property relationships, where thermal conditions influence intermolecular interactions and, consequently, separation efficiency and product composition (Figure 1b).

The purity of lecithin in this study ranged from 61.2% to 63.5%. The increase in purity may be attributed to improved separation of lecithin from oil and a reduction in sample moisture content. Conversely, the decrease in purity could be due to the degradation of certain lecithin compounds and an increased proportion of oil in the lecithin because of incomplete gum separation. Elevated water content during separation may also hinder gum removal, leading to the formation of three phases and a higher moisture content in the lecithin [26,27,28].

Sangkram and Noomhorm (2002) [29] reported that high processing temperatures increase impurities in lecithin, thereby reducing its recovery from soybean oil. Similarly, Srinuan et al. (2020) [21] reported values from 43.1% to 55.7%, lower than the values observed in the present study.

Previous studies have shown that increasing both temperature and water complicates the separation of lecithin from oil [22]. In addition, the potential enhancement of enzymatic hydrolysis under these conditions [30] may further contribute to reduce lecithin purity.

#### 2.1.3. Peroxide Value

Table 1 indicates that the quadratic model provided the best fit for the peroxide value data (*p* < 0.0001). According to the ANOVA results (Table 2), the linear effects of temperature and water addition, the quadratic effect of temperature, and the interaction between temperature and water addition significantly influenced the peroxide value of lecithin (*p* < 0.005). The peroxide value increased non-linearly with rising temperature and showed a slight increase with higher water addition (Figure 1c).

According to Table 3, the linear effect of temperature had the greatest influence on the peroxide value of lecithin. In this study, peroxide values ranged from 3.50 to 4.87 meq O_2_/kg. Elevated peroxide levels are known to alter the physicochemical and organoleptic properties of fats and oils, leading to the formation and accumulation of volatile aldehydes and ketones, which contribute to undesirable flavors and odors [31].

List et al. (1981) [16] reported that high temperatures during lecithin extraction cause darkening of lecithin, likely due to increased peroxide formation and the generation of additional compounds associated with higher temperatures and water content. The observed increase in peroxide value with rising temperature in this study may therefore be attributed to the enhanced oxidation of oils present in lecithin. Similarly, Souček et al. (2023) [32] demonstrated that even minor temperature fluctuation during oil filtration can increase peroxide values, which is consistent with the present findings [33]. The peroxide value of lecithin should not exceed 10 meq O_2_/kg; in this study, all treatments remained below this threshold [11,34]. Spessato et al. (2023) [35] reported that the method of lecithin decolorization from rice oil influenced peroxide values, with only one sample exceeding 10 meq O_2_/kg [29]. Rahman et al. (2011) [36] noted that factors such as temperature, humidity, light, heavy metals, and oxygen can affect peroxide formation. In particular, higher moisture content was associated with increased peroxide values, which is consistent with the results of this study.

#### 2.1.4. Acidy Number

The results of model fitting for the acidy number are presented in Table 1, indicating that the quadratic model provided the best fit (*p* = 0.0026). Statistical analysis showed that the linear effects of temperature and water addition, as well as the quadratic effect of water addition, significantly influenced the acidy number of lecithin (*p* < 0.01) (Table 2). Figure 1d illustrates the effects of temperature and water addition on the acidy number of lecithin. Both parameters showed a positive relationship, as increasing temperature and water addition resulted in higher acidy number s. As presented in Table 3, the linear effect of water addition was the most influential factor. In this study, the acidy number of lecithin ranged from 20.52 to 23.50 mg/g.

Srinuan et al. (2020) [21] reported the acidy number of soybean oil extracted with ethanol to be 21.8–31.9 mg/g. The acidy number reflects the concentration of free fatty acids formed mainly through hydrolytic reactions involving the cleavage of ester bonds in phospholipids and triglycerides. In the presence of moisture and elevated temperature, hydrolysis is accelerated, resulting in the release of free fatty acids and an increase in acidy number. Although linoleic acid is the predominant fatty acid in soybean lecithin and is susceptible to oxidative degradation, oxidation primarily leads to the formation of peroxides and secondary oxidation products rather than directly increasing the acidy number. Therefore, the increase in acidy number observed in this study is more likely associated with hydrolytic rancidity promoted by higher water addition and temperature. Similar observations were reported by Hidayah (2018) [37], who noted that increased moisture and temperature can enhance the release of free fatty acids.

In agreement with the findings of Hidayah (2018) [37], several factors such as temperature and moisture can contribute to an increase in acidy number through the release of free fatty acids. In the present study, both temperature and water addition showed significant linear effects on the acidy number (*p* < 0.01), while the quadratic effect of water addition was also significant (Table 1 and Table 2). As illustrated in Figure 1d, increasing temperature and water addition resulted in higher acidy number, indicating enhanced formation of free fatty acids. This trend is consistent with hydrolytic reactions, in which the presence of moisture and elevated temperature accelerates the cleavage of ester bonds in phospholipids and triglycerides. Therefore, the increase in acidy number observed in this study can be primarily attributed to hydrolytic rancidity promoted by higher water addition and temperature during processing.

### 2.2. Physicochemical Properties of Soybean Oil

#### 2.2.1. Acidity

The results of data fitting for soybean oil acidity are presented in Table 1, which show that the quadratic model provided the best fit (*p* = 0.0062). Statistical analysis indicated that the linear parameters of temperature and water addition, as well as the quadratic parameter of temperature, significantly influenced oil acidity (*p* < 0.005) (Table 2).

**Figure 2:**
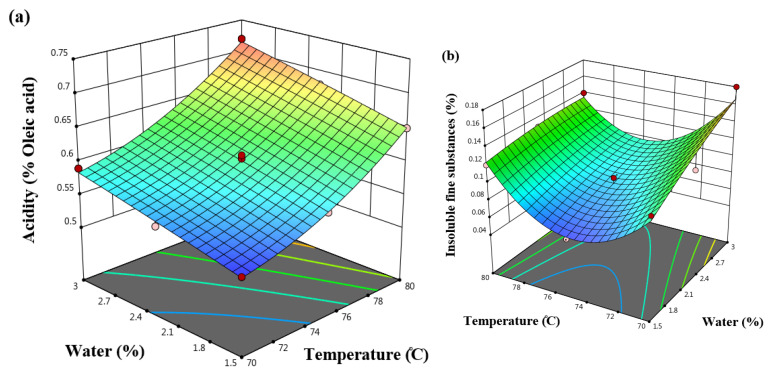
The effect of water and temperature on the (**a**) acidity and (**b**) insoluble fine substances of soybean oil.

Figure 2a illustrates the effects of temperature and water addition, revealing that higher temperatures resulted in increased soybean oil acidity, while additional water caused a slight increase. As shown in Table 3, the linear effect of temperature was the most influential factor. In this study, the acidity of soybean oil ranged from 0.53% to 0.72% (expressed as oleic acid).

The increase in acidity with rising temperature can be attributed to the chemical decomposition and hydrolysis of triglycerides resulting in the release of free fatty acids [29]. Bakhshabadi et al. (2017) [38] also reported that higher processing temperature and seed moisture content increased oil acidity during sunflower oil extraction. In the present study, the observed increase in oil acidity at higher temperature and water addition is therefore more likely related to enhanced hydrolytic reactions promoted by heat and moisture, which facilitate the cleavage of ester bonds in triglycerides and the formation of free fatty acids.

#### 2.2.2. Insoluble Fine Substances

Table 1 indicates that the quadratic model provided the best fit for assessing the effects of the parameters on insoluble fine substances. As shown in Table 2, the linear parameter of water addition and the quadratic parameter of temperature significantly influenced the insoluble fine substances in soybean oil (*p* < 0.01). According to the best-fit model, temperature had a greater impact on insoluble fine substances than the other parameters (Table 3). Increasing the amount of added water resulted in higher levels of insoluble fine substances in the soybean oil. In contrast, raising the temperature up to 75 °C reduced the amount of insoluble fine substances, but a further increase from 75 °C to 80 °C led to an increase (Figure 2b). Overall, the insoluble fine substances in soybean oil ranged from 0.04% to 1.17%.

Abad and Shahidi (2020) [39] developed a robust stripping method for removing minor components from edible oils, including phospholipid-associated impurities, and reported that it was more effective than conventional column chromatographic techniques.

Their findings highlight the importance of efficient separation processes in reducing residual minor compounds in the oil phase. Similarly, in the present study, the decrease in insoluble fine substances with increasing temperature up to 75 °C can be attributed to improved separator efficiency in removing lecithin-related compounds from the oil phase. However, further increases in temperature and moisture content appear to reduce the efficiency of lecithin separation, allowing more phospholipid-associated particles to remain in the oil phase and thereby increasing the level of insoluble fine substances.

Todorović et al. (2023) [40] demonstrated that elevated temperatures can promote chemical reactions among compounds, leading to the release of certain lecithin constituents, which may contribute to an increase in insoluble fine substances in the oil. Farzaneh et al. (2017) [41] also demonstrated that increasing the temperature and moisture content during oil extraction from rapeseeds results in a higher amount of fine substances insoluble in oil.

### 2.3. Optimizing the Lecithin Extraction on an Industrial Scale

The industrial-scale production of lecithin from soybean oil was optimized within a temperature range of 70–80 °C and a water addition of 1.5–3%. Optimal conditions were determined to maximize lecithin purity while minimizing moisture content, peroxide value, and acidy number, as well as to reduce acidity and insoluble fine substances in the soybean oil. According to the results, the best conditions for lecithin production were 73.93 °C and 1.5% water (R^2^ = 0.874). The optimal temperature of 73.93 °C obtained from the RSM model represents a theoretical value. In industrial practice, such conditions can be effectively implemented within a practical operating range (approximately 72–75 °C), considering standard process control tolerances and the relatively low sensitivity of the system to minor temperature variations. The overall desirability value (0.874) was calculated using the desirability function approach, in which each response was assigned equal importance and transformed into a dimensionless desirability function. Global desirability was computed as the geometric mean of individual desirability, reflecting the simultaneous optimization of all responses.

**Figure 3:**
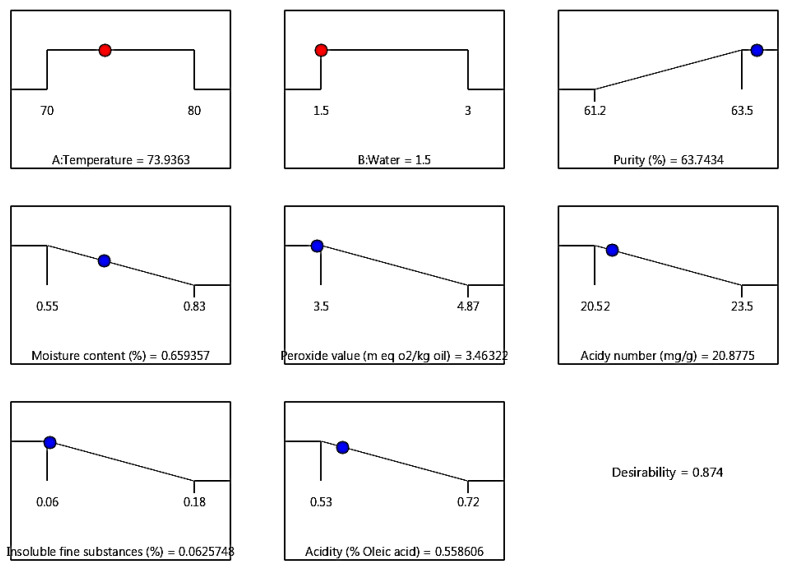
The data of lecithin production obtained from the software under optimal conditions.

To validate the predictive models, the experimental data obtained under these optimal conditions were compared with the model-predicted values for all measured characteristics (Table 3; Figure 3). No significant differences were observed between the predicted and actual values, confirming the reliability of the models.

## 3. Materials and Methods

### 3.1. Raw Materials

Soybeans, the sole raw material used in this study, were sourced from Golestan Province, Iran. Upon arrival at the processing facility, the raw soybeans were transferred to storage silos prior to processing.

At the initial stage, impurities and foreign materials—including stones, plant debris, damaged seeds, and other extraneous matter—were removed using an industrial winnowing and cleaning system. This system operates based on a combination of air flow and mechanical screening to separate lighter impurities (e.g., dust, chaff) from heavier particles and intact soybeans.

This pre-cleaning step is essential to ensure uniform raw material quality, prevent equipment damage, and improve the efficiency of subsequent processing operations (Andreotti Impianti, Rome, Italy).

### 3.2. Oil Extraction

To prepare the soybeans for oil extraction, they were first fed into a cracker machine (Buhler, Uzwil, Switzerland, capacity of 500 tons per day) and crushed into smaller particles. The crushed particles were then transferred to a cooking pot (Andreotti, Calenzano, Italy, capacity of 500 tons per day), where processing conditions were adjusted to achieve an output temperature of 60 °C and a moisture content of 12%.

Following conditioning, the material was passed through a flaking machine (Buhler, Switzerland) to produce uniform soybean flakes with increased surface area, facilitating efficient solvent extraction. The flakes were subsequently subjected to solvent extraction using hexane in a continuous extractor (Desmet–Andreotti, Rome, Italy) at 55 °C for 7 h.

The resulting liquid phase (miscella), consisting of hexane and dissolved soybean oil, was filtered to remove residual fine particles. Oil recovery was then carried out by heating the miscella in a desolventizer–toaster–dryer–cooler (DTDC) unit, where hexane was evaporated and separated, yielding crude soybean oil (Andreotti, Italy) [42].

### 3.3. Lecithin Extraction and Drying

The extracted crude soybean oil was heated to 70 °C prior to hydration. Subsequently, water (1.5–3% *w*/*w* of oil) preheated to 70–80 °C was added, and the mixture was subjected to continuous agitation for 30 min to promote phospholipid hydration and aggregation.

The lecithin production line was equipped with a temperature control system (Andreotti, Rome, Italy) and a flowmeter (Krohne, H250, Duisburg, Germany) to accurately monitor and regulate water temperature and dosage. All processing parameters were controlled through an automated system operated from the central monitoring unit.

Following hydration, the mixture was transferred to a centrifugal separator (Westfalia, Oelde, Germany) operating at 4000 rpm, where phase separation occurred, yielding a lecithin-rich phase and a degummed soybean oil phase. The recovered lecithin was subsequently dried to a final moisture content below 1% to ensure product stability and prevent microbial or oxidative deterioration [43].

### 3.4. Moisture Content

Moisture content was determined according to the AOCS (44-15) method [21,43,44]. A measured quantity of lecithin was heated in a laboratory oven (Memmert, Schwabach, Germany) at 100 °C until a constant weight was obtained. The percentage of weight loss during this process was recorded as the moisture content.

### 3.5. Insoluble Fine Substances in the Oil

The content of insoluble fine substances in the oil was determined by centrifuging approximately 10 mL of soybean oil in centrifuge tubes (Thermo, Tokyo, Japan) at 4000 rpm for 10 min. The weight of sediment collected at the bottom of the tube was multiplied by 10 and expressed as the percentage of insoluble fine substances [45,46].

### 3.6. Purity

First, 10 g of lecithin was weighed and washed three times with 100 mL of acetone. To obtain pure phosphatides, a saturated phosphatide-acetone solution was prepared and maintained at 5 °C. After 2 h at this temperature, the solution was filtered, and the pure phosphatides were collected. Approximately 5 g of purified phosphatides were dissolved in petroleum ether. Subsequently, 25 mL of acetone was added to the solution, which was centrifuged at 2000 rpm for 5 min. The centrifuge tubes were then placed in a cold bath for 15 min. The sediment formed was collected as pure phosphatide.

The washed lecithin was heated in water to 60 °C. Approximately 2 g of the warmed lecithin was transferred into a centrifuge tube containing 15 mL of the phosphatide-acetone solution and heated until the lecithin was completely melted. The tube was then placed in a cold bath (5 °C for 5 min). The contents were diluted to 40 mL with cold phosphatide-acetone solution and centrifuged at 2000 rpm for 5 min. The sedimented phase was separated and allowed to stand at room temperature to evaporate residual acetone. The material was subsequently dried in an oven, and the purity percentage was calculated according to Equation (1) [46].

(1) $$P=\frac{R\times 100}{S}-\;B,$$

where *P* is purity (%), *R* is the sediment weight (g), *S* is the sample weight, and *B* represents the insoluble fine substances in toluene.

### 3.7. Acidy Numberand Acidity

The acidy number of soybean oil and acidity of lecithin (expressed as oleic acid) were determined according to standard procedure [47]. A 5 g sample of oil was mixed with 25 mL of ethanol, and a few drops of phenolphthalein were added as an indicator. The mixture was titrated with 0.1 N of KOH until a persistent purple color appeared [41,48]. The acidy number and acidity were calculated using Equations (2) and (3):

(2) $$Acid\;Value=\frac{V\times N\times 56.4}{W},$$

(3) $$Acidity=\frac{282\times N\times 100\times V}{1000\times W},$$

in which *V* is the volume of NaOH solution (mL), *N* is the normality of NaOH, and *W* is the sample weight (g).

### 3.8. Peroxide Value

The peroxide value of lecithin was determined using the AOCS Cd 8-53 method [49]. A solvent mixture of acetic acid and chloroform (3:2, *v*/*v*) was prepared; then, 5 g of soybean oil were added to 300 mL of this solvent and mixed thoroughly. Next, 0.5 mL of saturated potassium iodide solution was added, and the mixture was kept in darkness for 1 min. Afterward, 30 mL of distilled water was added, and the solution was titrated with 0.1 M sodium thiosulfate until the blue color disappeared [50]. The peroxide value was calculated according to Equation (4):

(4) $$P=\frac{S\times M\times 100}{W},$$

where *P* is peroxide value (meq O_2_/kg sample), *S* is the volume of sodium thiosulfate (mL), *M* is the molarity of sodium thiosulfate (mol/L), and *W* is the sample weight (g).

### 3.9. Statistical Analysis

Response surface methodology (RSM) was employed to evaluate the effects of extraction parameters, namely inlet soybean oil temperature (70, 75, and 80 °C) and water content (1.5%, 2.25%, and 3%), on the physicochemical properties of lecithin (moisture content, purity, peroxide value, and acidy number) and outlet soybean oil (acidity and insoluble substances) using a central composite rotatable design (CCRD). The use of RSM was selected to examine factor interactions and identify optimal conditions for lecithin and soybean oil production. Four models (linear, two-factor interaction (2FI), quadratic, and cubic) were applied to fit the data. Statistical analysis was performed using Design-Expert software, version 13 (Stat-Ease Inc., Minneapolis, MN, USA). All experiments were carried out in five replications at the central point.

## 4. Conclusions

This study provides both scientific and practical insights into the optimization of industrial-scale soybean lecithin production using response surface methodology (RSM). From a scientific perspective, the results elucidate the combined effects of temperature and water addition on key physicochemical properties, thereby contributing to a deeper understanding of process–structure–property relationships governing lecithin separation systems. The developed regression models, supported by high coefficients of determination and acceptable variability, confirm the robustness and predictive capability of the applied optimization approach.

From an industrial standpoint, the findings demonstrate that high-quality lecithin can be consistently produced at approximately 74 °C with 1.5% water addition. Considering typical process control tolerances, these conditions can be reliably implemented within a practical operating window (e.g., 72–75 °C), ensuring process stability without requiring excessive precision. Furthermore, the results clearly indicate that excessive temperature or water addition adversely affects both lecithin quality and oil characteristics, thereby defining critical operational boundaries for industrial applications.

Despite these contributions, certain limitations should be acknowledged. The experimental design was restricted to a defined range of process variables and focused primarily on two factors, while other potentially influential parameters—such as mixing intensity, residence time, and raw material variability—were not investigated. In addition, although the statistical models effectively describe system behavior, the underlying mechanistic interactions governing phase separation and lecithin composition were not explored in detail.

Future research should aim to enhance process robustness by incorporating additional operational variables and validating the proposed models under diverse industrial conditions and feedstock variations. Moreover, in-depth mechanistic investigations into emulsion stability, phospholipid behavior, and phase separation dynamics are recommended to further strengthen the scientific foundation of the process. Such efforts would support improved process control, facilitate scale-up, and expand industrial applicability.

## Figures and Tables

**Table 1:** Model selection for dependent variables.

Variable/Model											
		LSS	*p*-Value	2FISS	*p*-Value	QSS	*p*-Value	CSS	*p*-Value	RSS	TSS
Lecithin	MC (%)	0.0550	<0.0001	0.0001	0.3060	0.0001	0.5590	0.0003	0.1637	0.0003	6.38
	P (%)	1.26	0.4029	0.1225	0.6827	5.72	0.0001	0.3683	0.0163	0.0880	51,114.33
	PV (meq O_2_/kg oil)	1.59	0.0005	0.0342	0.4153	0.4087	<0.0001	0.0128	0.0013	0.0010	201.58
	AV (mg/g)	7.55	<0.0001	0.0576	0.3689	0.4740	0.0026	0.0150	0.6815	0.0905	6448.90
Soybean oil	IFSs (%)	0.0021	0.3890	0.0006	0.4590	0.0083	0.0006	0.0004	0.3200	0.0007	0.1586
	A (%Oleic acid)	0.0295	<0.0001	0.0000	0.7036	0.0011	0.0062	0.0001	0.4964	0.0003	4.87

MC = Moisture Content, P = Purity, PV = Peroxide Value, AV = Acidy number, IFSs = Insoluble Fine Substances, A = Acidity, LSS = Linear Sum of Square, 2FISS = 2FI Sum of Square, QSS = Quadratic Sum of Square, CSS = Cubic Sum of Square, RSS = Residual Sum of Square, TSS = Total Sum of Square.

**Table 2:** Analysis of variance for determined parameters *.

Variable			Model	X_1_	X_2_	X_1×2_	X_1_^2^	X_2_^2^	Residual	Pure Error	Cor. Total
Lecithin	MC (%)	SS	0.0550	0.0368	0.0181	-	-	-	0.0009	0.0003	0.0558
		*p*-Value	<0.0001	<0.0001	<0.0001	-	-	-	-	-	-
	P (%)	SS	7.10	0.0417	1.22	0.1225	4.83	0.0014	0.4564	0.0280	7.56
		*p*-Value	0.0004	0.4503	0.0035	0.2128	<0.0001	0.8881	-	-	-
	PV (m eq o_2_/kg oil)	SS	2.04	1.35	0.2400	0.0342	0.3742	0.0038	0.0137	0.0007	2.05
		*p*-Value	<0.0001	<0.0001	<0.0001	0.0041	<0.0001	0.2086	-	-	-
	AV (mg/g)	SS	8.08	1.85	5.70	0.0576	0.0277	0.4639	0.1055	0.0565	8.19
		*p*-Value	<0.0001	<0.0001	<0.0001	0.0915	0.2171	0.0009	-	-	-
Soybean oil	IFSs (%)	SS	0.0110	0.0001	0.0020	0.0006	0.0074	0.0000	0.0011	0.0001	0.0121
		*p*-Value	0.0018	0.5424	0.0097	0.0907	0.0003	0.6822	-	-	-
	A (% Oleic acid)	SS	0.0306	0.0241	0.0054	0.0000	0.0010	2.956E-06	0.0003	0.0001	0.0309
		*p*-Value	<0.0001	<0.0001	<0.0001	0.4970	0.0027	0.8125	-	-	-

* X_1_: Temperature, X_2_: Water. MC = Moisture Content, P = Purity, PV = Peroxide Value, AV = Acidy number, IFSs = Insoluble Fine Substances A = Acidity, SS = Sum of Square.

**Table 3:** Designed equation models for dependent variables *.

Dependent Variable		Equation	R^2^	R^2^-Adjusted	CV %	Real Data
Lecithin	MC (%)	y = +0.6977 − 0.0783x_1_ + 0.0550x_2_	0.9890	0.9811	1.14	0.661
	P (%)	y = +63.32 − 0.0833x_1_ − 0.4500x_2_ + 0.1750x_1×2_ − 1.32x_1_^2^ − 0.0224x_2_^2^	0.9396	0.8965	0.4072	63.762
	PV (m eq o_2_/kg oil)	y = 3.76 + 0.4750x_1_ + 0.20x_2_ + 0.0925x_1×2_ + 0.3681x_1_^2^ − 0.0369x_2_^2^	0.9933	0.9885	1.13	3.471
	AV (mg/g)	y = +22.40 + 0.5550x_1_ + 0.9750x_2_ − 0.1200x_1×2_ + 0.1002x_1_^2^ − 0.4098x_2_^2^	0.9871	0.9779	0.5515	20.796
Soybean oil	IFSs (%)	y = +0.0838 − 0.0033x_1_ + 0.0183x_2_ + −0.0125x_1×2_ + 0.0517x_1_^2^ − 0.0033x_2_	0.9060	0.8388	12.01	0.060
	A (% Oleic acid)	y = +0.6017 + 0.0633x_1_ + 0.0300x_2_ + 0.0025x_1×2_ + 0.0190x_1_^2^ − 0.0010x_2_^2^	0.9890	0.9811	1.14	0.562

* X_1_: Temperature, X_2_: Water. MC = Moisture Content, P = Purity, PV = Peroxide Value, AV = Acidy number, IFSs = Insoluble Fine Substances, A = Acidity. Note: The higher CV for insoluble fine substances is due to their low concentration and sensitivity to process variations under industrial-scale conditions.

## Data Availability

No data was used for the research described in the article.
